# Prevalence Patterns of Avian *Plasmodium* and *Haemoproteus* Parasites and the Influence of Host Relative Abundance in Southern China

**DOI:** 10.1371/journal.pone.0099501

**Published:** 2014-06-09

**Authors:** Yanhua Zhang, Yuchun Wu, Qiang Zhang, Dongdong Su, Fasheng Zou

**Affiliations:** 1 South China Botanical Garden, Chinese Academy of Sciences, Guangzhou, China; 2 University of Chinese Academy of Sciences, Beijing, China; 3 Guangdong Entomological Institute/South China Institute of Endangered Animals, Guangzhou, China; Centro de Pesquisa Rene Rachou/Fundação Oswaldo Cruz (Fiocruz-Minas), Brazil

## Abstract

Infectious diseases threaten the health and survival of wildlife populations. Consequently, relationships between host diversity, host abundance, and parasite infection are important aspects of disease ecology and conservation research. Here, we report on the prevalence patterns of avian *Plasmodium* and *Haemoproteus* infections and host relative abundance influence based on sampling 728 wild-caught birds representing 124 species at seven geographically widespread sites in southern China. The overall prevalence of two haemoprotozoan parasites, *Plasmodium* and *Haemoproteus*, was 29.5%, with 22.0% attributable to *Haemoproteus* and 7.8% to *Plasmodium*. *Haemoproteus* prevalence differed significantly among different avian host families, with the highest prevalence in Nectariniidae, Pycnonotidae and Muscicapidae, whereas *Plasmodium* prevalence varied significantly among host species. Seventy-nine mitochondrial lineages including 25 from *Plasmodium* and 54 from *Haemoproteus* were identified, 80% of which were described here for the first time. The phylogenetic relationships among these parasites indicated stronger host-species specificity for *Haemoproteus* than *Plasmodium*. Well-supported host-family (Timaliidae) specific clades were found in both *Plasmodium* and *Haemoproteus*. The *Haemoproteus* tree shows regional subclades, whereas the *Plasmodium* clades are “scattered” among different geographical regions. Interestingly, there were statistically significant variations in the prevalence of *Plasmodium* and *Haemoproteus* among the geographical regions. Furthermore, the prevalence of *Plasmodium* and *Haemoproteus* were not significantly correlated with host relative abundance. Further efforts will focus on exploring the relationships between parasite prevalence and sex, age, and immune defense of the host.

## Introduction

*Plasmodium* and *Haemoproteus* (Phylum Apicomplexa, class Haemosporida) are common vector-borne globally distributed blood parasites, which occur in most bird species [1], [2]. Such haemosporidian parasites are closely related genetically but differ in their life cycles and their primary transmission vectors [3]. The parasites reproduce asexually in the vertebrate host and sexually in a dipteran vector [1], [2]. *Plasmodium* is transmitted primarily by mosquitoes (genera *Culex*, *Aedes* and *Culiseta*), while *Haemoproteus* is transmitted by biting midges (Ceratopogonidae) and louse flies (Hippoboscidae) [1], [2]. These parasites are commonly used as model systems for testing hypotheses in evolutionary ecology [4], [5] and for investigating diagnostic traits and control options for human malaria [6]. Although the symptoms of infection with haematozoa are generally mild in birds, such parasites can affect avian body condition [7], reproductive success [8], community structure [9] and possibly lead to host extinction [10]. Consequently, these parasites can exert strong selective forces on their hosts, making it important to gain a better understanding of their distribution, dispersal potential, and host specificity in wild bird populations.

Recently, the development of molecular genetic screening techniques for avian blood parasites has revealed many novel aspects of their ecology, including much higher than expected levels of diversity [11], dispersal by migratory birds [12], parasite host specificity [13], phylogenetic relationships [14] and the complexity of host–parasite relationships [15]. However, the majority of the ecological studies on haemosporidian parasites have not considered the possibility that parasite prevalence and lineage distribution may vary with host abundance. Current hypotheses predict divergent outcomes for relationship between host diversity and parasite prevalence. For example, the “Dilution Effect” hypothesis predicts that high host diversity will reduce the relative number of susceptible hosts, and reduce encounters between susceptible and infected hosts, thereby resulting in lower parasite prevalence [16]. In contrast, the “Amplification Effect” hypothesis predicts that high host diversity will increase susceptible host number, increase encounters between susceptible and infected hosts, or through the presence of secondary hosts, which will result in high parasite prevalence [17].

Therefore, we have molecularly characterized the lineage diversity and distribution of *Plasmodium* and *Haemoproteus* in bird communities from southern China, where limited data are available on avian haemosporidia, and have inferred the phylogenetic relationships between these parasites. In particular, we have examined whether prevalence in haemosporidian parasites are related to host relative abundance. Characterization and demonstration of the haemosporidian parasites communities presented herein are the first step towards further investigations of host-parasite systems in tropical environments.

## Materials and Methods

### Ethics statement

This study was conducted according to protocols approved by the Administrative Panel on Laboratory Animal Care (approval number 2009001) of South China Institute of Endangered Animals. Moreover,all field studies was approved by the State Forestry Administration, China,which is the authority that issued the permit for each location in this study.

### Study sites, relative abundance estimation, and sampling

**Figure 1:**
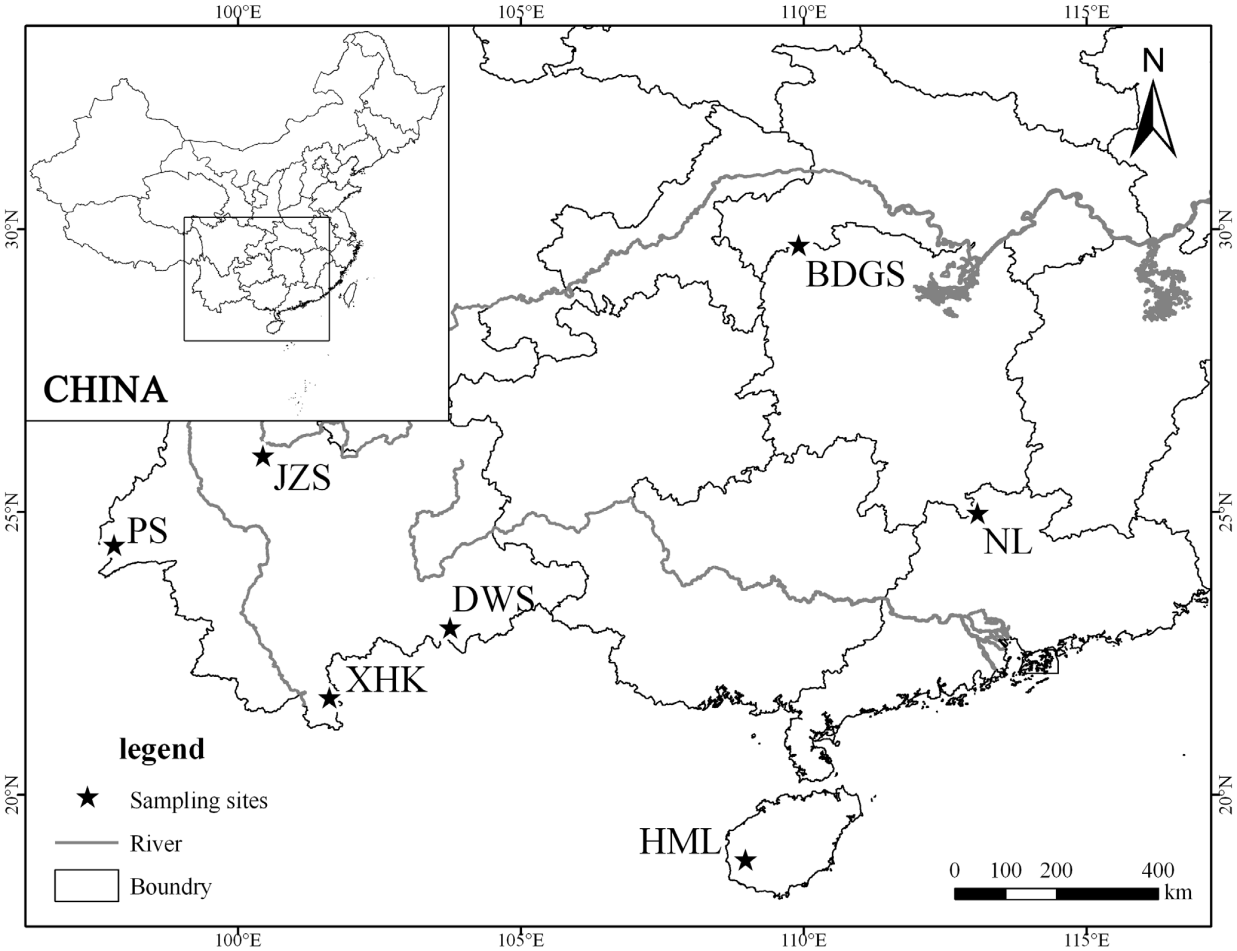
Map of southern China showing sampling sites. Sampling site names are coded: BDGS, Badagongshan; NL, Nanling; HML, Houmiling; XHK, Xinhuikuan; JZS, Jizushan; DWS, Daweishan; PS, Pingshan.

This study was carried out between July 2012 and July 2013 at seven distinct areas across the southern China region (Table 1, Figure 1). Repeated samplings were conducted in Jizushan (twice), Badagongshan (three times), and Nanling (three times). We sampled the birds using mist nets. Two connected mist nets were installed where vegetation and topography permitted, and all nets were situated in the forest interior. The distance separating the nets at the same sampling site was more than 200 m [18]. Mist net (12 m long, 2.6 m high, four-shelf nets; 10–14 nets placed systematically per plot and run simultaneously) capture was carried out for 5–6 consecutive days without rain or strong wind for each period per plot, from 06:30 to 17:30 each day. Nets were checked and individual captured birds were ringed, measured and released, and we recorded the shelf on which the birds were captured. A small amount of blood (approximately 10 µl) was collected by puncturing the brachial vein with a small needle. The blood was preserved in 96% ethanol at −20°C [19], [20]. Host relative abundance was calculated by the mean capture rate (individuals per 100 net-hours) [21], [22]. The capture rate method, as a measure of relative abundance, is controversial because the rates from this type of capture can be affected by factors such as weather, net location, net tension, habitat structure, as well as the vertical movement, flight distance, and flight frequency of individuals [21], [23], [24]. However, mist netting has many distinct advantages over point counts: it can be readily standardized, is relatively free from observer biases, and species that are difficult to see and seldom vocalize can be sampled [25], [26]. Mist-netting results have been validated by comparison with other techniques, and its use is recommended over other methods for collecting data on tropical understory avifauna [22].

**Table 1:** Summary data for each sampling site.

Site	N	No. infected	*Plasmodium* (%)	*Haemoproteus* (%)	Host relative abundance (mean±SE; individuals/100.net-hours) *	Lineage richness
Badagongshan-Tianpingshan Station	16	1	6.25	0.00	2.13±0.93 (9)	1
Badagongshan-Chaye Station	21	6	23.81	4.76	3.37±0.98 (16)	4
Badagongshan-Wudaoshuizhen Station	61	0	0.00	0.00	4.70±0.78 (45)	0
Nanling (Nov. 2012)	68	4	4.41	1.47	9.47±1.59 (62)	4
Nanling (July. 2012)	23	1	4.35	0.00	6.95±2.00 (18)	1
Nanling (Apr. 2013)	58	6	1.72	8.62	13.06±2.82 (50)	6
Houmiling	75	20	8.00	18.67	3.97±0.65 (55)	11
Xinhuikuan	89	65	21.35	52.81	10.83±1.73 (72)	24
Jizushan (Aug. 2013)	77	20	7.79	19.48	9.18±1.20 (67)	8
Jizushan (Sept. 2013)	37	9	10.81	13.51	2.75±0.63 (28)	7
Daweishan	53	6	3.77	7.55	6.39±1.33 (47)	5
Pingshan	150	77	6.00	45.33	18.54±3.31 (136)	24
Total	728	215	7.80	22.00	7.77±0.50 (605)	79

* Numbers in brackets representing sampling numbers of birds captured only used for analyzing the relationship between the parasites prevalence and host relative abundance.

### DNA extraction, amplification and sequencing

DNA was extracted from the blood samples of 728 birds using the TIANamp Genomic DNA Kit (Beijing, China), following the manufacturer's guidelines. All of the extracted DNA samples were screened for parasite infections using a highly efficient nested PCR that amplifies a partial segment of the mitochondrial cytochrome *b* (*cyt b*) gene of *Plasmodium* and *Haemoproteus* parasites following the methods described by Hellgren et al. [27]. To detect false positives, two negative controls (ddH_2_0) were included for each set of 24 samples, as well as a positive control comprising an avian blood sample that was known to be from a parasite-infected individual. Positive or negative infections were seen as the presence or absence of bands of approximately 500 bp on 2% agarose gels using 6 µl of each PCR product. Additionally, PCR-negative infections were confirmed by repeating the PCR. All PCR-positive samples were sequenced from the 5′-end using HaemF primers [20]. To ensure that the DNA extractions were successful for those samples in which we did not detect an infection by PCR, we amplified the second subunit of the avian nicotinamide adenine dinucleotide dehydrogenase gene (*ND2*) using L5215 and H6313 primers, according to the methods of Johnson and Sorenson [28]. This second amplification was successful for all of the samples analyzed herein.

### Sequence analysis and phylogenetic reconstruction

Sequences were assembled and aligned by eye using SeqMan 7.1.0 (DNAStar Inc., Madison, WI, USA). Parasites with sequences differing by one nucleotide substitution were considered to represent evolutionarily independent lineages [29], and sequences with double peaks were considered mixed infections. We treated mixed infections as separate events (i.e., a double infection was considered resolved only when it yielded a match with a single pair of previously identified lineages and no double peaks were left unexplained) following the method Perez-Tris and Bensch [30]. All unsolved mixed infections were withdrawn from the data set. Lineages were identified by comparison with published sequences available at GenBank (http://www.ncbi.nlm.nih.gov/genbank/), and named according to the MalAvi Public Database (http://mbio-serv2.mbioekol.lu.se/Malavi/index.html). Lineages that were not present in the MalAvi database were considered to be new lineages.

Phylogenetic analyses were performed separately from alignments that consisted of 342 bp *cyt b Haemoproteus* sequences and 459 bp *cyt b Plasmodium* sequences after removal of redundant sequences [31]. We first determined the model of sequence evolution that best fitted the data using MODELTEST version.3.7 [32]. Bayesian analysis of the sequence data was then conducted with MRBAYES version 3.1.2 [33] using the model of sequence evolution obtained from MODELTEST. Two Markov chains were run simultaneously for 6 million generations and with sampling every 100 generations. The first 15,000 trees (25%) were discarded as “burn-in” and the remaining trees were used to calculate the posterior probabilities.

### Statistical analyses

We calculated capture rate of understory birds as a measure of host relative abundance after omitting the following species: 1) species more than 250 g in weight, 2) canopy-feeding frugivores, nectivores, and carnivores, and 3) rare species where only one individual was recorded [34]. Accordingly, a total of 605 individuals representing 75 species in 20 families were used to analyze the relationship between the parasites prevalence and host relative abundance (Table 1). All data were examined for normality using Kolmogorov-Smirnov tests. To assess variations in host relative abundance among regions, and whether parasite prevalence differed across host species (included ≥7 individuals), host families (considered ≥5 species, ≥19 individuals) [35], [36], and geographical regions, we used K independent sample nonparametric tests. To assess differences in host and region specialization of avian Haemosporidia, we used Mann–Whitney U tests or independent samples T tests. Relationship between the prevalence of haemosporidian parasites and host relative abundance was determined by Spearman correlation analyses. Statistical analyses were performed with SPSS 19.0.

## Results

### Parasite prevalence

Among 728 bird samples representing 124 species from 26 families that were screened for infection with *Plasmodium* or *Haemoproteus*, we detected an overall prevalence of 29.5% (215 positive samples) comprising 71 infected species from 18 families (Table S1). Of these, 7.8% representing 34 species were infected with *Plasmodium*, while 22.0% representing 58 species were infected with *Haemoproteus*.

**Figure 2:**
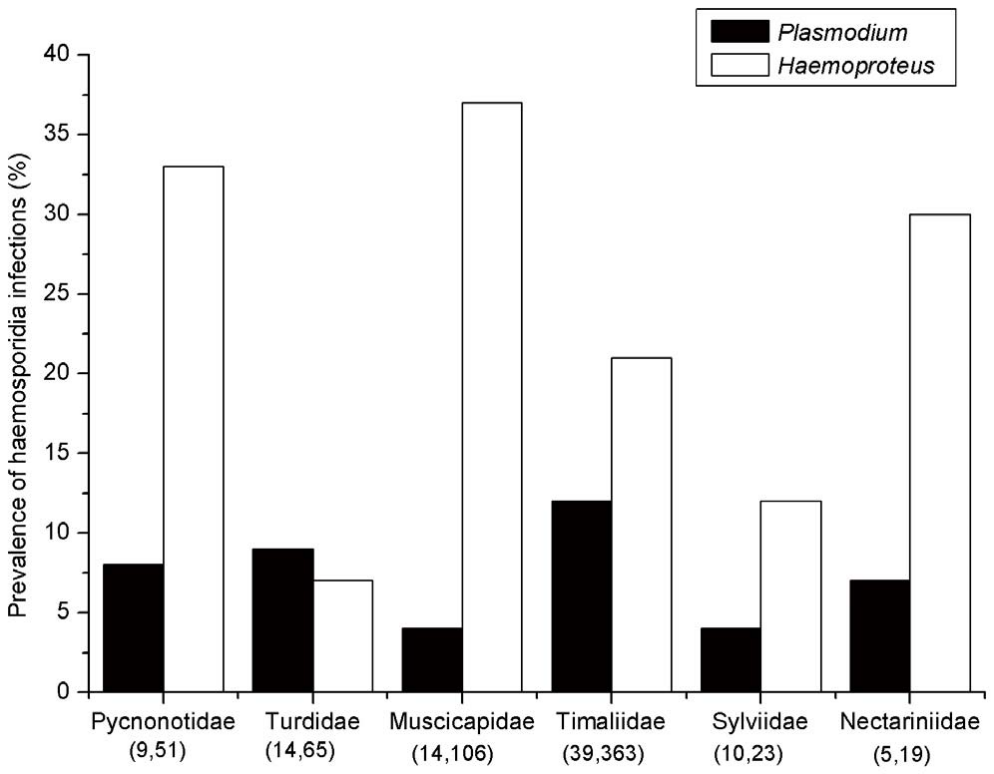
Prevalence of haemosporidian infections in selected avian host families from Southern China. Number of species and individuals per family are shown in brackets. *Plasmodium* prevalence was not significantly different among families (*X* ^2^ = 1.122, *df* = 5, *p* = 0.952). *Haemoproteus* prevalence was significantly different among families (*X^2^* = 13.104, *df* = 5, *p* = 0.022).

We found that the *Plasmodium* prevalence varied significantly among sampled avian species (*X* ^2^ = 46.956, *df* = 31, *p* = 0.033), with Rufous-capped Babbler (*Stachyris nigriceps*) having the highest prevalence (60%). In contrast, the prevalence of *Haemoproteus* did not differ significantly among sampled avian species (*X^2^ = *32.890, *df* = 31, *p* = 0.375). Among families, the prevalence of *Haemoproteus*, which ranged from 7% in the Turdidae to 37% in Muscicapidae, was not uniform (*X^2^* = 13.104, *df* = 5, *p* = 0.022;Figure 2). The prevalence of *Plasmodium* was relatively low, and no significant difference was evident among avian families (*X* ^2^ = 1.122, *df* = 5, *p* = 0.952; Figure 2). The family assignment and frequency of parasite detection for all host species examined are listed in Table S1.

### Parasite lineages

Overall, cytochrome *b* gene sequencing revealed 25 *Plasmodium* and 54 *Haemoproteus* lineages (GenBank accession numbers are listed in Table S2: KJ145047- KJ145125); most of these lineages were recorded for the first time in this study. The lineage richness among the sites ranged from 0 to 24 lineages.

**Figure 3:**
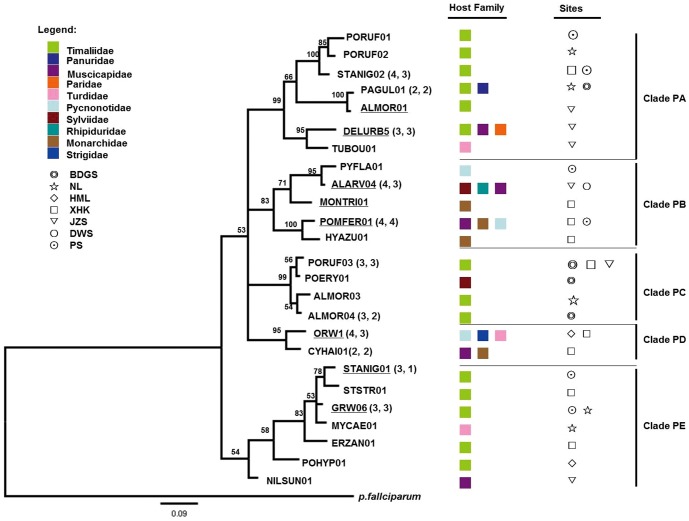
Phylogenetic relationships among *Plasmodium cyt b* lineages. Outgroup: human malaria parasite *P. falciparum*. Numbers located near branches indicate the Bayesian probability values. Previously described lineages are underlined. Lineages recovered from more than one individual are indicated. The number of individuals and host species in each lineage is shown in brackets. Survey sites are coded: BDGS, Badagongshan; NL, Nanling; HML, Houmiling; XHK, Xinhuikuan; JZS, Jizushan; DWS, Daweishan; PS, Pingshan.

**Figure 4:**
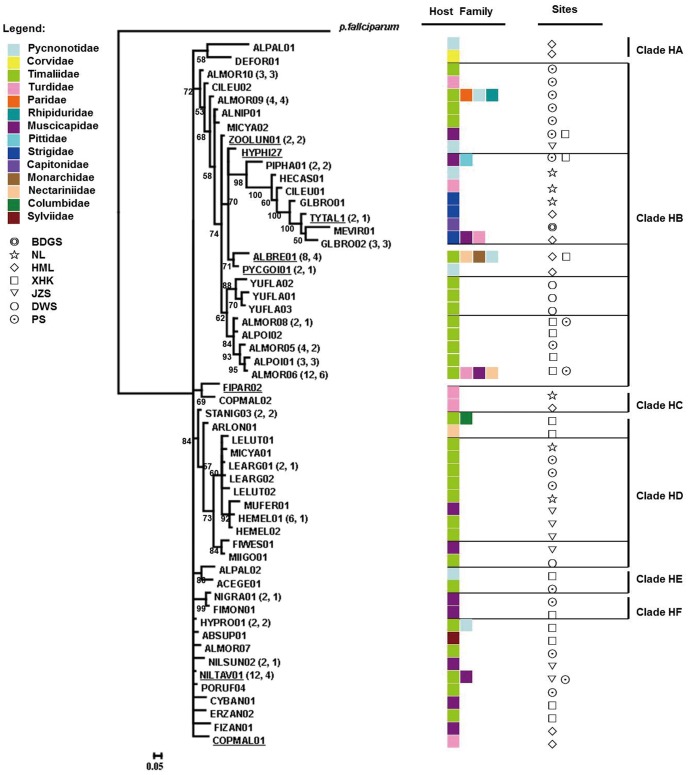
Phylogenetic relationships among *Haemoproteus cyt b* lineages. Outgroup: human malaria parasite *P. falciparum*. Numbers located near branches indicate the Bayesian probability values. Previously described lineages are underlined. Lineages recovered from more than one individual are indicated. The number of individuals and host species in each lineage is shown in brackets. Survey sites are coded: BDGS, Badagongshan; NL, Nanling; HML, Houmiling; XHK, Xinhuikuan; JZS, Jizushan; DWS, Daweishan; PS, Pingshan.

The host range of *Plasmodium* lineages varied from a single species to four host species, whereas *Haemoproteus* lineages presented a wider range, varying from one to six host species (Figures 3, 4). Moreover, the most frequent *Haemoproteus* lineage (ALMOR06) was also the most widespread among the sampling sites and host families, which was identified in four avian families and six host species (Table S2, Figure 4). Another highly frequent *Haemoproteus* lineage (NILTAV01) was recorded in two avian families and four host species. The number of parasite genetic lineages in each host species varied between one and 11 (Table S1). The host with the greatest number of lineages recorded (11 in total) was the Grey-cheeked Fulvetta (*Alcippe morrisonia*); this value probably reflects the larger sample size for this species (Table S1). The prevalence of *Plasmodium* and *Haemoproteus* parasites in this host species was 3.2% and 26.4%, respectively. Additionally, most of these parasite lineages were positioned closely in the phylogenetic tree (Figure 3: GRW06, ALMOR03, ALMOR04; Figure 4: ALMOR05, ALPOI01, NILTAV01, ALMOR06, ALMOR07, ALMOR08, ALMOR09, ALMOR10). However, the ALBRE01 and HEMEL01 lineages that were observed in a single host species (*Alophoixus pallidus* and *Heterophasia melanoleuca*, respectively), which were recorded more than five times, should be viewed with caution (Table S2).

*Plasmodium* and *Haemoproteus* mitochondrial lineage relationships are presented independently in Figures 3 and 4, respectively. The *Plasmodium* lineages appear in two different groups: both of these groups are clustered among haplotypes that infect hosts of different taxonomic affiliations. Within *Haemoproteus*, our data could not resolve deep hierarchical relationships, which resulted in a large basal polytomy interspersed with haplotypes infecting many different host species spanning the whole diversity of avian *Haemoproteus*.

The proportion of parasite lineages sharing identical sequences in more than one host species occurs more frequently (Mann–Whitney U Test: *Z* = 2.562, *p = *0.010) in *Plasmodium* than in *Haemoproteus*. This may indicate higher rates of host-switching and reduced host specificity in *Plasmodium* (see Figures 3 and 4 for details of the host species). Again, significant specificity in parasite lineages was observed at the host-family level. For example, one host family (Timaliidae) has well-supported host-family specific *Plasmodium* clades (clade PA, clade PE; Figure 3), as well as *Haemoproteus* clades (one subclade of clade HB and clade HD; Figure 4). Within the *Haemoproteus* tree (Figure 4), there were cladeHA, subclades of clade HB and clade HD shared almost the same geographic regions. In *Plasmodium* (Figure 3), there was no shared clade. *Haemoproteus* shows a significant degree of regional fidelity (Independent Samples T Test:*t* = 4.575, *df* = 10, *p = *0.001), whereas *Plasmodium* does not.

### The relationship between parasite prevalence and host relative abundance

There was significant difference in the prevalence of *Plasmodium* (*X* ^2^ = 23.109, *df* = 11, *p* = 0.017) among regions, and a highly significant difference for the prevalence of *Haemoproteus* among regions was detected (*X* ^2^ = 54.307, *df* = 11, *p*<0.001). The bird relative abundance (mean ± SE; individuals per 100 net-hours) was the highest in Pingshan (18.54±3.31), and lowest in Badagongshan-Tianpingshan Station (2.13±0.93). Moreover, the host relative abundance also varied significantly among areas (*X* ^2^ = 74.821, *df* = 11, *p*<0.001; Table 1). In the Spearman correlation analyses, regional variations in *Plasmodium* and *Haemoproteus* infections were not significantly associated with differences in the host relative abundance of the local areas (*r* = 0.168, *p* = 0.601 for *Plasmodium*; *r* = 0.437, *p* = 0.156 for *Haemoproteus*).

## Discussion

### Parasite prevalence

*Plasmodium* and *Haemoproteus* are widely distributed blood parasites that appear to be nearly ubiquitous in avian communities. The southern China region harbours a diverse community of avian haematozoan lineages that were distributed among 57.3% (71/124) of the birds sampled in this study. We estimated an overall parasite prevalence of about 29.5%. Estimates for the prevalence of haemoprotozoan parasites in bird populations from tropical regions are about 12% in Costa Rica by blood smear [37], 33% in the central Philippine islands [38], 35% in Neotropical Brazil [19], 40% in Central Africa [39], and 50% in India [35]. Clearly, a high prevalence of avian haematozoa was found in southern China as that recorded in many other areas in the world. In addition, we found that the prevalence of *Haemoproteus* in birds was higher than that seen for *Plasmodium*. This finding is consistent with previous studies, which showed that *Haemoproteus* was generally more prevalent than *Plasmodium* [38], [40], [41]. This difference might be explained by the fact that *Haemoproteus* has lower pathogenicity in its host than *Plasmodium* [10], [42]. Heavily infected birds, at the peak of their infections, are seldom sampled using mist nets because they are probably less mobile or active than healthy individuals [2]. Another reason for the difference in prevalence of *Plasmodium* and *Haemoproteus* may relate to the abundances of vector populations that transmit these parasites [1], [2].

In our study, the prevalence of *Haemoproteus* varied significantly among host families; these parasites were most prevalent in Nectariniidae, Pycnonotidae and Muscicapidae birds (Figure 2). A plausible explanation for the variation in parasites observed in the different bird families could relate to a vector preference for certain species of birds coupled with the ability of the parasite to complete its development in a given host [38]. Furthermore, Nectariniidae and Pycnonotidae are tropical birds. Generally, tropical zones have a higher prevalence of parasite relapse infections, as well as increased vector abundance and decreased host immunocompetance [43]. In the present study, most Muscicapidae were captured in the first shelf, suggesting that they have an inclination to forage in the bush close to the ground where haemosporidian vectors tend to be more abundant [13]. Silva-Iturriza et al. [38] also inferred in their study conducted in Asia that Pycnonotidae and Muscicapidae are prone to high levels of *Haemoproteus* infection. However, prevalence estimates, even when accurate, should be considered snapshots in time and space [44].

### Parasite lineages

Sixty-three parasite lineages, representing 79.7% of all the recorded lineages, are reported here for the first time. This result indicates that the sites sampled should be of special interest to researchers studying the infection patterns and species distribution of haemoprotozoan parasites in birds. Wide variation was observed in the number of hosts from which we recovered individual parasite lineages, which ranged from one to six avian host species. The ALMOR06 lineage (*Haemoproteus* sp.), which was first identified in the present study, exhibited the greatest local abundance and host diversity (Table S2). Another frequently encountered lineage, NILTAV01 (*Haemoproteus* sp.), which was first recorded in Vivid Niltava (*Niltava vivida*), was isolated from Myanmar [35]. In our study, we identified NILTAV01 in more than one avian host species (Table S2).

In the parasite phylogenies (Figures 3, 4), more *Plasmodium* lineages are shared by multiple host species than that observed for *Haemoproteus*. Several studies have shown a lack of host fidelity in *Plasmodium* infections [38], [45], [46]. This indicates that host-switching between avian species is more likely to occur in *Plasmodium* than in *Haemoproteus*, which historically, could be caused by greater host fidelity among *Haemoproteus*-transmitting vectors (hippoboscid and ceratopogonid flies) than *Plasmodium*-transmitting mosquitoes [1], [47]. Mosquito vectors are generalist blood feeders that are likely to transmit parasites to multiple avian species [48]. Importantly, wide variability in haemosporidian parasites host specificity may be linked to wide variation in parasite virulence [49]. In fact, specialists presumably benefit from the relatively high fitness conferred by parasitizing a limited number of hosts, and may, therefore, be able to evolve more quickly in response to changes in host defence or physiology. Generalists, however, may be less prone to extinction because they maintain larger population sizes distributed over a greater number of hosts [50]. We found one well-supported host-family (Timaliidae) specific clade, not only in *Haemoproteus*, but also in *Plasmodium*. Babblers (family Timaliidae), are an especially important component of the tropical Asian avifauna [51], [52], and are an indicator of the health of forest environments in southern China [53]. So, at least some lineages of *Plasmodium* appear to be constrained to certain host groups to the same extent as *Haemoproteus* lineages. Thus, the strategy adopted by a parasite represents a fine balance between the selective pressures favouring either specialist or generalist approaches. But the signals of host specificity that extend deeper within the *Haemoproteus* phylogeny suggest that many of these lineages are likely to be true specialists.

Several studies have investigated the geographical distribution of genetically distinct avian haemosporidian parasites in different geographical regions and habitats [19], [39], [54], [55]. In our phylogenetic trees (Figures 3, 4), the *Haemoproteus* tree shows regional subclades and a significant level of region-specific fidelity. Thus, *Haemoproteus* appears to have a high affiliation with a single bird fauna and a single transmission area. Contrastingly, *Plasmodium* clades are “scattered” among geographical regions, thereby making the proportion of *Plasmodium* lineages able to be transmitted higher than that of *Haemoproteus* in our study sites. *Plasmodium* parasites transmit more often, as is the case for SGS1 [56], which is the most prevalent of all *Plasmodium* lineages, infecting hosts from over a dozen different avian families in distinct continents [57]. Differences in the geographic distribution of parasite lineages at the different sampling sites are dependent on the distribution of host species and may be explainable by the fact that different parasite lineages are associated with particular vector communities [58] or habitats [19], [54], [59]; these predictions should be tested in-depth study.

### Parasite prevalence and host relative abundance association

Host abundance influences the infection dynamics of parasites [60]. Host abundance is often positively correlated with parasite prevalence. For example, positive associations were found between abundance of host birds and the prevalence and abundance of trematode parasites in snail populations [61], [62]. In addition, models of tick-borne zoonoses often predict that disease risk is positively correlated with host diversity, as long as high diversity leads to high total abundance of hosts [63]. Nevertheless, avian malaria parasites prevalence assumed a U-shaped distribution with respect to host abundance [9].

The effects of host abundance on disease prevalence may be explained by a few non-mutually exclusive hypotheses. The greater prevalence observed among more abundant avian hosts is consistent with high host-to-vector transmission rates (and overall higher encounter rates) in dense populations [64], [65], [66], when pathogen transmission is frequency-dependent [16]. A higher numbers of susceptible hosts (high competency hosts) within a community will result in an increase in parasite prevalence and in the risk of infection through “Amplification Effects” [16], [17]. For example, in Hawaii, parasite transmission is maintained by susceptible native birds and a disease reservoir of chronically infected native birds [11]. High bird abundance may also reduce the availability of food or other resources, resulting in more intense competition, thereby suppressing the immune systems of such birds [67]. In the present study, infections with *Plasmodium* and *Haemoproteus* were not associated with host relative abundance. It is likely that vector-related factors such as vector competence and distribution are important for their transmission [68]. In fact, a very complex relationship exists between the host and parasite. To fully understand the impact of host abundance and how abundance variations impact coevolutionary interactions, our future study efforts will focus on exploring the extent to which parasite prevalence is affected by differences in sex, age, immune defence of the host.

## Supporting Information

Table S1 **Total number of individuals sampled and frequency of infections with** ***Plasmodium*** **(P) and** ***Haemoproteus*** **(H), including the number of lineages recorded in a host.**
(DOC)

Table S2 **Summary of lineage parasite information, including Genbank accession numbers.**
(DOC)

## References

[1] Atkinson CT, van Riper C III (1991) Pathogenicity and epizootiology of avian haematozoa: *Plasmodium*, *Leucocytozoon*, and *Haemoproteus*. In: Loye JE, Zuk M `(Eds.) Bird-parasite interactions: ecology, evolution, and behaviour. Oxford, Oxford University Press. pp. 19–48.

[2] Valkiunas G (2005) Avian malaria parasites and other haemosporidia: CRC Press, Boca Raton, Florida.

[3] Martinsen ES, Perkins SL, Schall JJ (2008) A three-genome phylogeny of malaria parasites (*Plasmodium* and closely related genera): evolution of life-history traits and host switches. Molecular Phylogenetics and Evolution 47: 261–273. 18248741 10.1016/j.ympev.2007.11.012

[4] Ricklefs RE, Fallon SM, Bermingham E (2004) Evolutionary relationships, cospeciation, and host switching in avian malaria parasites. Systematic Biology 53: 111–119. 14965906 10.1080/10635150490264987

[5] Knowles SC, Nakagawa S, Sheldon BC (2009) Elevated reproductive effort increases blood parasitaemia and decreases immune function in birds: a meta-regression approach. Functional Ecology 23: 405–415.

[6] Slater LB (2005) Malarial birds: Modeling infectious human disease in animals. Bulletin of the History of Medicine 79: 261–294. 15965289 10.1353/bhm.2005.0092

[7] Valkiūnas G, Zickus T, Shapoval AP, Iezhova TA (2006) Effect of *Haemoproteus belopolskyi* (Haemosporida: Haemoproteidae) on body mass of the blackcap *Sylvia atricapilla* . Journal of Parasitology 92: 1123–1125. 17152968 10.1645/GE-3564-RN.1

[8] Tomás G, Merino S, Moreno J, Morales J, Martínez-de La Puente J (2007) Impact of blood parasites on immunoglobulin level and parental effort: a medication field experiment on a wild passerine. Functional Ecology 21: 125–133.

[9] Ricklefs RE, Swanson BL, Fallon SM, MartÍnez-AbraÍn A, Scheuerlein A, et al (2005) Community relationships of avian malaria parasites in southern Missouri. Ecological Monographs 75: 543–559.

[10] Atkinson CT, Dusek RJ, Woods KL, Iko WM (2000) Pathogenicity of avian malaria in experimentally-infected Hawaii Amakihi. Journal of Wildlife Diseases 36: 197–204. 10813599 10.7589/0090-3558-36.2.197

[11] LaPointe DA, Atkinson CT, Samuel MD (2012) Ecology and conservation biology of avian malaria. Annals of the New York Academy of Sciences 1249: 211–226. 22320256 10.1111/j.1749-6632.2011.06431.x

[12] Loiseau C, Harrigan RJ, Cornel AJ, Guers SL, Dodge M, et al (2012) First evidence and predictions of *Plasmodium* transmission in Alaskan bird populations. PLoS One 7: e44729. 23028595 10.1371/journal.pone.0044729 PMC3446979

[13] Svensson-Coelho M, Blake JG, Loiselle BA, Penrose AS, Parker PG, et al (2013) Diversity, prevalence, and host specificity of avian *Plasmodium* and *Haemoproteus* in a Western Amazon assemblage. Ornithological Monographs 76: 1–47.

[14] Tania J, Gavin H, Olof H, Ian P (2012) Migratory behavior of birds affects their coevolutionary relationship with blood parasites. Evolution 66: 740–751. 22380437 10.1111/j.1558-5646.2011.01470.x

[15] Rigaud T, Perrot-Minnot M-J, Brown MJ (2010) Parasite and host assemblages: embracing the reality will improve our knowledge of parasite transmission and virulence. Proceedings of the Royal Society B: Biological Sciences 277: 3693–3702. 20667874 10.1098/rspb.2010.1163 PMC2992712

[16] Keesing F, Holt RD, Ostfeld RS (2006) Effects of species diversity on disease risk. Ecology Letters 9: 485–498. 16623733 10.1111/j.1461-0248.2006.00885.x

[17] Ostfeld RS, LoGiudice K (2003) Community disassembly, biodiversity loss, and the erosion of an ecosystem service. Ecology 84: 1421–1427.

[18] Zou FS, Chen GZ, Yang QF, Li YD (2012) Bird species richness along an elevational gradient in forest at Jianfengling, Hainan Island, China. Zoological Studies 51: 362–371.

[19] Lacorte GA, Félix GM, Pinheiro RR, Chaves AV, Almeida-Neto G, et al (2013) Exploring the diversity and distribution of neotropical avian malaria parasites-a molecular survey from southeast Brazil. PLoS One 8: e57770. 23469235 10.1371/journal.pone.0057770 PMC3585926

[20] Marzal A, Bensch S, Reviriego M, Balbontin J, De Lope F (2008) Effects of malaria double infection in birds: one plus one is not two. Journal of Evolutionary Biology 21: 979–987. 18462316 10.1111/j.1420-9101.2008.01545.x

[21] Remsen Jr J, Good DA (1996) Misuse of data from mist-net captures to assess relative abundance in bird populations. The Auk 113: 381–398.

[22] Dunn EH, Ralph CJ (2004) Use of mist nets as a tool for bird population monitoring. Studies in Avian Biology 29: 1–6.

[23] Karr JR (1981) Surveying birds with mist nets. Studies in Avian Biology 6: 62–67.

[24] MacArthur RH, MacArthur AT (1974) On the use of mist nets for population studies of birds. Proceedings of the National Academy of Sciences 71: 3230–3233. 10.1073/pnas.71.8.3230 PMC388657 16578721

[25] Terborgh J, Robinson SK, Parker III TA, Munn CA, Pierpont N (1990) Structure and organization of an Amazonian forest bird community. Ecology Monograph 60: 213–238.

[26] Blake JG, Loiselle BA (2001) Bird assemblages in second-growth and old-growth forests, Costa Rica: perspectives from mist nets and point counts. The Auk 118: 304–326.

[27] Hellgren O, Waldenström J, Bensch S (2004) A new PCR assay for simultaneous studies of *Leucocytozoon*, *Plasmodium*, and *Haemoproteus* from avian blood. The Journal of Parasitology 90: 797. 15357072 10.1645/GE-184R1

[28] Johnson K, Sorenson M (1998) Comparing molecular evolution in two mitochondrial protein coding genes (cytochrome *b* and *ND2*) in the dabbling ducks (Tribe: Anatini). Molecular Phylogenetics and Evolution 10: 82–94. 9751919 10.1006/mpev.1997.0481

[29] Bensch S, Péarez-Tris J, Waldenströum J, Hellgren O (2004) Linkage between nuclear and mitochondrial DNA sequences in avian malaria parasites: Multiple cases of cryptic speciation? Evolution 58: 1617–1621. 15341164 10.1111/j.0014-3820.2004.tb01742.x

[30] Perez-Tris J, Bensch S (2005) Diagnosing genetically diverse avian malarial infections using mixed-sequence analysis and TA-cloning. Parasitology-Cambridge 131: 15–24. 10.1017/s003118200500733x 16038392

[31] Cornuault J, Bataillard A, Warren BH, Lootvoet A, Mirleau P, et al (2012) The role of immigration and in-situ radiation in explaining blood parasite assemblages in an island bird clade. Molecular Ecology 21: 1438–1452. 22332752 10.1111/j.1365-294X.2012.05483.x

[32] Posada D, Crandall KA (1998) Modeltest: testing the model of DNA substitution. Bioinformatics 14: 817–818. 9918953 10.1093/bioinformatics/14.9.817

[33] Huelsenbeck JP, Ronquist F (2001) MRBAYES: Bayesian inference of phylogenetic trees. Bioinformatics 17: 754–755. 11524383 10.1093/bioinformatics/17.8.754

[34] Srinivasan U (2013) A slippery slope: logging alters mass–abundance scaling in ecological communities. Journal of Applied Ecology 50: 920–928.

[35] Ishtiaq F, Gering E, Rappole JH, Rahmani AR, Jhala YV, et al (2007) Prevalence and diversity of avian hematozoan parasites in Asia: a regional survey. Journal of Wildlife Diseases 43: 382–398. 17699077 10.7589/0090-3558-43.3.382

[36] Beadell JS, Gering E, Austin J, Dumbacher JP, Peirce MA, et al (2004) Prevalence and differential host-specificity of two avian blood parasite genera in the Australo-Papuan region. Molecular Ecology 13: 3829–3844. 15548295 10.1111/j.1365-294X.2004.02363.x

[37] Valkiūnas G, Iezhova TA, Brooks DR, Hanelt B, Brant SV, et al (2004) Additional observations on blood parasites of birds in Costa Rica. Journal of Wildlife Diseases 40: 555–561. 15465725 10.7589/0090-3558-40.3.555

[38] Silva-Iturriza A, Ketmaier V, Tiedemann R (2012) Prevalence of avian haemosporidian parasites and their host fidelity in the central Philippine islands. Parasitology International 61: 650–657. 22819957 10.1016/j.parint.2012.07.003

[39] Ricklefs RE, Fallon SM (2002) Diversification and host switching in avian malaria parasites. Proceedings of the Royal Society of London Series B: Biological Sciences 269: 885–892. 12028770 10.1098/rspb.2001.1940 PMC1690983

[40] Belo N, Rodríguez-Ferraro A, Braga E, Ricklefs R (2012) Diversity of avian haemosporidians in arid zones of northern Venezuela. Parasitology 139: 1021–1028. 22405405 10.1017/S003118201200039X

[41] Møller AP, Nielsen JT (2007) Malaria and risk of predation: a comparative study of birds. Ecology 88: 871–881. 17536704 10.1890/06-0747

[42] van Riper III C, van Riper SG, Goff ML, Laird M (1986) The epizootiology and ecological significance of malaria in Hawaiian land birds. Ecological Monographs 56: 327–344.

[43] Durrant KL, Beadell JS, Ishtiaq F, Graves GR, Olson SL, et al (2006) Avian hematozoa in South America: a comparison of temperate and tropical zones. Ornithological Monographs: 98–111.

[44] Loiseau C, Harrigan RJ, Bichet C, Julliard R, Garnier S, et al (2013) Predictions of avian *Plasmodium* expansion under climate change. Scientific Reports 3: 1126 doi: 10.1038/srep01126 23350033 10.1038/srep01126 PMC3553554

[45] Beadell JS, Covas R, Gebhard C, Ishtiaq F, Melo M, et al (2009) Host associations and evolutionary relationships of avian blood parasites from West Africa. International Journal for Parasitology 39: 257–266. 18713636 10.1016/j.ijpara.2008.06.005 PMC2632718

[46] Waldenström J, Bensch S, Kiboi S, Hasselquist D, Ottosson U (2002) Cross-species infection of blood parasites between resident and migratory songbirds in Africa. Molecular Ecology 11: 1545–1554. 12144673 10.1046/j.1365-294x.2002.01523.x

[47] Bennett GF, Bishop MA, Peirce MA (1993) Checklist of the avian species of *Plasmodium* Marchiafava & Celli, 1885 (Apicomplexa) and their distribution by avian family and Wallacean life zones. Systematic Parasitology 26: 171–179.

[48] Hellgren O, Pérez-Tris J, Bensch S (2009) A jack-of-all-trades and still a master of some: prevalence and host range in avian malaria and related blood parasites. Ecology 90: 2840–2849. 19886492 10.1890/08-1059.1

[49] Garamszegi, Zsolt L (2006) The evolution of virulence and host specialization in malaria parasites of primates. Ecology Letters 9: 933–940. 16913936 10.1111/j.1461-0248.2006.00936.x

[50] Woolhouse ME, Taylor LH, Haydon DT (2001) Population biology of multihost pathogens. Science 292: 1109–1112. 11352066 10.1126/science.1059026

[51] Cibois A, Hackett S (2003) Mitochondrial DNA phylogeny of babblers (Timaliidae). The Auk 120: 35–54.

[52] Moyle RG, Andersen MJ, Oliveros CH, Steinheimer FD, Reddy S (2012) Phylogeny and biogeography of the core babblers (Aves: Timaliidae). Systematic Biology 61: 631–651. 22328569 10.1093/sysbio/sys027

[53] Zhang Q, Zou FS, Zhang M, Huang JH (2010) The distribution pattern of the babblers (Timaliidae) in China. Acta Zootaxonomica Sinica 35: 135–144 (In Chinese with English summary)..

[54] Wood MJ, Cosgrove CL, Wilkin TA, Knowles SC, Day KP, et al (2007) Within-population variation in prevalence and lineage distribution of avian malaria in blue tits, *Cyanistes caeruleus* . Molecular Ecology 16: 3263–3273. 17651202 10.1111/j.1365-294X.2007.03362.x

[55] Chasar A, Loiseau C, Valkiūnas G, Iezhova T, Smith T, et al (2009) Prevalence and diversity patterns of avian blood parasites in degraded African rainforest habitats. Molecular Ecology 18: 4121–4133. 19754513 10.1111/j.1365-294X.2009.04346.x

[56] Hellgren O, Waldenström J, Peréz-Tris J, Ösi E, Hasselquist D, et al (2007) Detecting shifts of transmission areas in avian blood parasites—a phylogenetic approach. Molecular Ecology 16: 1281–1290. 17391413 10.1111/j.1365-294X.2007.03227.x

[57] Hellgren O, Kutzer M, Bensch S, Valkiūnas G, Palinauskas V (2013) Identification and characterization of the merozoite surface protein 1 (*msp1*) gene in a host-generalist avian malaria parasite, *Plasmodium relictum* (lineages SGS1 and GRW4) with the use of blood transcriptome. Malaria Journal 12: 381. 24172200 10.1186/1475-2875-12-381 PMC3827925

[58] Hellgren O (2005) The occurrence of haemosporidian parasites in the Fennoscandian bluethroat (*Luscinia svecica*) population. Journal of Ornithology 146: 55–60.

[59] Loiseau C, Harrigan RJ, Robert A, Bowie RC, Thomassen HA, et al (2012) Host and habitat specialization of avian malaria in Africa. Molecular Ecology 21: 431–441. 22142265 10.1111/j.1365-294X.2011.05341.x PMC3253197

[60] Johnson PT, Preston DL, Hoverman JT, Richgels KL (2013) Biodiversity decreases disease through predictable changes in host community competence. Nature 494: 230–233. 23407539 10.1038/nature11883

[61] Hechinger RF, Lafferty KD (2005) Host diversity begets parasite diversity: bird final hosts and trematodes in snail intermediate hosts. Proceedings of the Royal Society B: Biological Sciences 272: 1059–1066. 16024365 10.1098/rspb.2005.3070 PMC1599879

[62] Fredensborg BL, Mouritsen KN, Poulin R (2006) Relating bird host distribution and spatial heterogeneity in trematode infections in an intertidal snail—from small to large scale. Marine Biology 149: 275–283.

[63] Dobson A (2004) Population dynamics of pathogens with multiple host species. The American Naturalist 164: S64–S78. 10.1086/424681 15540143

[64] Brown CR, Komar N, Quick SB, Sethi RA, Panella NA, et al (2001) Arbovirus infection increases with group size. Proceedings of the Royal Society of London Series B: Biological Sciences 268: 1833–1840. 11522203 10.1098/rspb.2001.1749 PMC1088816

[65] Galvani AP (2003) Epidemiology meets evolutionary ecology. Trends in Ecology and Evolution 18: 132–139.

[66] Wonham MJ, de-Camino-Beck T, Lewis MA (2004) An epidemiological model for West Nile virus: invasion analysis and control applications. Proceedings of the Royal Society of London Series B: Biological Sciences 271: 501–507. 15129960 10.1098/rspb.2003.2608 PMC1691622

[67] Tracy CR, Nussear K, Esque T, Dean-Bradley K, Tracy C, et al (2006) The importance of physiological ecology in conservation biology. Integrative and Comparative Biology 46: 1191–1205. 21672817 10.1093/icb/icl054

[68] Kimura M, Dhondt AA, Lovette IJ (2006) Phylogeographic structuring of *Plasmodium* lineages across the North American range of the house finch (*Carpodacus mexicanus*). Journal of Parasitology 92: 1043–1049. 17152948 10.1645/GE-639R.1

