# Correction: Prevalence Patterns of Avian *Plasmodium* and *Haemoproteus* Parasites and the Influence of Host Relative Abundance in Southern China

**DOI:** 10.1371/journal.pone.0107826

**Published:** 2014-09-03

**Authors:** The PLOS ONE Staff

There is an error in the first sentence of the second paragraph of the Results. The correct sentence is: We found that the *Plasmodium* prevalence varied significantly among sampled avian species (*X* ^2^  =  46.956, *df*  =  31, *p*  =  0.033), with Black-headed Babbler (*Stachyris nigriceps*) having the highest prevalence (60%).

There are errors in Table S1 and Table S2. In both Supplementary Tables, the species name *Hypsipetes propinquus aquilonis* should read *Iole propinqua*. Please see the correct Tables S1 and S2 below.

## Supporting Information

Table S1 **Total number of individuals sampled and frequency of infections with** ***Plasmodium*** **(P) and** ***Haemoproteus*** **(H), including the number of lineages recorded in a host.**
(DOC)

Table S2 **Summary of lineage parasite information, including Genbank accession numbers.**
(DOC)
