# Supplementary material for: Correction: Prevalence Patterns of Avian Plasmodium and Haemoproteus Parasites and the Influence of Host Relative Abundance in Southern China
Source: PLoS One. 2014 Sep 3;9(9):e107826. doi: 10.1371/journal.pone.0107826 (PMC4153723; doi:10.1371/journal.pone.0107826)
Supplement: Table S1 — Total number of individuals sampled and frequency of infections with Plasmodium (P) and Haemoproteus (H), including the number of lineages recorded in a host. (DOC) [file pone.0107826.s001.doc]

**Table S1.** Total number of individuals sampled and frequency of infections with *Plasmodium* (P) and *Haemoproteus* (H), including the number of lineages recorded in a host.

| **Family** | **Species** | **Common Name** | **Total** | **No. of birds infected ( P%** **(mean±SE）)** | | **No. of lineages** |
| --- | --- | --- | --- | --- | --- | --- |
|  |  |  |  | **P** | **H** |  |
| Columbidae | *Chalcophaps indica* | Emerald Dove | 2 | 0 | 1 | 1 |
| Cuculidae | *Clamator coromandus* | Red-winged Crested Cuckoo | 1 | 0 | 0 | 0 |
|  | *Cuculus saturatus* | Oriental Cuckoo | 1 | 0 | 0 | 0 |
|  | *Surniculus lugubris* | Drongo Cuckoo | 1 | 0 | 0 | 0 |
| Strigidae | *Otus bakkamoena* | Collared Scops Owl | 4 | 2 | 2 | 2 |
|  | *Glaucidium brodiei* | Collared Pygmy Owl | 2 | 0 | 2 | 2 |
| Trogonidae | *Harpactes erythrocephalus* | Red-headed Trogon | 1 | 0 | 0 | 0 |
| Capitonidae | *Megalaima virens* | Great Barbet | 1 | 0 | 1 | 1 |
| Picidae | *Picumnus innominatus* | Speckled Piculet | 9 | 0（0.00） | 1（0.11±0.11） | 0 |
|  | *Sasia ochracea* | White-browed Rufous Piculet | 1 | 1 | 1 | 0 |
|  | *Blythipicus pyrrhotis* | Yellow-billed Bay Woodpecker | 2 | 0 | 0 | 0 |
| Eurylaimidae | *Serilophus lunatus* | Silver-breasted Broadbill | 7 | 1（0.14） | 1（0.14） | 0 |
| Pittidae | *Pitta phayrei* | Eared Pitta | 1 | 0 | 1 | 1 |
| Campephagidae | *Hemipus picatus* | Bar-winged Flycatcher Shrike | 2 | 0 | 0 | 0 |
| Pycnonotidae | *Spizixos canifrons* | Crested Finchbill | 1 | 0 | 0 | 0 |
|  | *Spizixos semitorques* | Collared Finchbill | 5 | 0 | 0 | 0 |
|  | *Pycnonotus sinensis* | Chinese Bulbul | 9 | 1（0.11） | 2（0.22） | 2 |
|  | *Pycnonotus flavescens* | Flavescent Green Bulbul | 2 | 1 | 1 | 1 |
|  | *Alophoixus pallidus* | White-throated Bulbul | 12 | 0（0.00） | 8（0.67±0.34） | 3 |
|  | *Iole propinqua* | Grey-eyed Bulbul | 1 | 0 | 1 | 1 |
|  | *Hemixos castanonotus* | Chestnut Bulbul | 6 | 0 | 1 | 1 |
|  | *Hypsipetes mcclellandii* | Green-winged Bulbul | 12 | 1（0.06±0.06） | 1（0.06±0.06） | 2 |
|  | *Hypsipetes leucocephalus* | Black Bulbul | 3 | 0 | 1 | 1 |
| Laniidae | *Lanius tigrinus* | Tiger Shrike | 1 | 1 | 0 | 0 |
| Dicruridae | *Dicrurus paradiseus* | Greater Racket-tailed Drongo | 2 | 0 | 0 | 0 |
| Corvidae | *Urocissa erythrorhyncha* | Red-billed Blue Magpie | 1 | 0 | 0 | 0 |
|  | *Dendrocitta formosae* | Gray Treepie | 1 | 0 | 1 | 1 |
| Turdidae | *Luscinia cyane* | Siberian Blue Robin | 1 | 0 | 0 | 0 |
|  | *Tarsiger cyanurus* | Red-flanked Bush Robin | 9 | 0（0.00） | 1（0.11） | 1 |
|  | *Copsychus malabaricus* | White-rumped Shama | 12 | 0（0.00） | 3（0.25） | 3 |
|  | *Cinclidium leucurum* | White-tailed Blue Robin | 17 | 0（0.00） | 5（0.47±0.19） | 3 |
|  | *Enicurus scouleri* | Little Forktail | 1 | 0 | 0 | 0 |
|  | *Enicurus immaculatus* | Black-backed Forktail | 1 | 0 | 0 | 0 |
|  | *Enicurus schistaceus* | Slaty-backed Forktail | 1 | 0 | 0 | 0 |
|  | *Enicurus leschenaulti* | White-crowned Forktail | 3 | 0 | 1 | 1 |
|  | *Enicurus maculatus* | Spotted Forktail | 4 | 0 | 0 | 0 |
|  | *Monticola rufiventris* | Chestnut-bellied Rock Thrush | 1 | 0 | 0 | 0 |
|  | *Myophonus caeruleus* | Blue Whistling Thrush | 2 | 1 | 0 | 1 |
|  | *Zoothera citrina* | Orange-headed Ground Thrush | 10 | 2（0.32±0.19） | 0（0.00） | 1 |
|  | *Turdus hortulorum* | Grey-backed Thrush | 1 | 0 | 0 | 0 |
|  | *Turdus boulboul* | Grey-winged Blackbird | 2 | 1 | 0 | 1 |
| Muscicapidae | *Rhinomyias brunneata* | Brown-chested Jungle Flycatcher | 5 | 0 | 0 | 0 |
|  | *Muscicapa ferruginea* | Ferruginous Flycatcher | 3 | 0 | 1 | 1 |
|  | *Ficedula zanthopygia* | Yellow-rumped Flycatcher | 2 | 0 | 1 | 1 |
|  | *Ficedula mugimaki* | Robin Flycatcher | 1 | 0 | 0 | 0 |
|  | *Ficedula hyperythra* | Snowy-browed Flycatcher | 16 | 1（0.04±0.04） | 0（0.00） | 1 |
|  | *Ficedula monileger* | White-gorgetted Flycatcher | 8 | 1（0.17±0.17） | 3（0.44±0.24） | 3 |
|  | *Ficedula westermanni* | Little Pied Flycatcher | 1 | 0 | 1 | 1 |
|  | *Niltava grandis* | Large Niltava | 3 | 0 | 3 | 2 |
|  | *Niltava macgregoriae* | Small Niltava | 13 | 0（0.00） | 1（0.17±0.17） | 1 |
|  | *Niltava davidi* | Fujian Niltava | 3 | 0 | 2 | 1 |
|  | *Niltava sundara* | Rufous-bellied Niltava | 23 | 3（0.20±0.15） | 8（0.13±0.13） | 4 |
|  | *Cyornis hainanus* | Hainan Blue Flycatcher | 12 | 1（0.33±0.33） | 2（0.07±0.07） | 2 |
|  | *Cyornis banyumas* | Hill Blue Flycatcher | 5 | 1 | 2 | 3 |
|  | *Culicicapa ceylonensis* | Grey-headed Canary Flycatcher | 11 | 0（0.00） | 3（0.50±0.29） | 0 |
| Rhipiduridae | *Rhipidura hypoxantha* | Yellow-bellied Fantail | 1 | 1 | 0 | 1 |
|  | *Rhipidura albicollis* | White-throated Fantail | 12 | 0（0.00） | 2（0.08±0.08） | 1 |
| Monarchidae | *Hypothymis azurea* | Black-naped Monarch | 6 | 4 | 1 | 4 |
|  | *Terpsiphone paradisi* | Asian Paradise Flycatcher | 2 | 0 | 1 | 1 |
| Timaliidae | *Garrulax albogularis* | White-throated Laughingthrush | 1 | 0 | 0 | 0 |
|  | *Garrulax monileger* | Lesser Necklaced Laughingthrush | 5 | 0 | 0 | 0 |
|  | *Garrulax maesi* | Grey Laughingthrush | 2 | 0 | 0 | 0 |
|  | *Garrulax chinensis* | Black-throated Laughingthrush | 1 | 0 | 0 | 0 |
|  | *Garrulax cineraceus* | Ashy Lauthingthrush | 1 | 0 | 0 | 0 |
|  | *Garrulax caerulatus* | Grey-sided Laughingthrush | 1 | 0 | 0 | 0 |
|  | *Garrulax canorus* | Hwamei | 7 | 0（0.00） | 0（0.00） | 0 |
|  | *Liocichla phoenicea* | Crimson-winged Liocichla | 1 | 0 | 0 | 0 |
|  | *Pellorneum albiventre* | White-bellied Jungle Babbler | 12 | 2（0.11±0.11） | 5（0.28±0.28） | 4 |
|  | *Pellorneum ruficeps* | Streak-breasted Jungle Babbler | 1 | 0 | 1 | 0 |
|  | *Pomatorhinus hypoleucos* | Large Scimitar Babbler | 1 | 1 | 0 | 1 |
|  | *Pomatorhinus erythrogenys* | Rusty-cheeked Scimitar Babbler | 1 | 0 | 0 | 0 |
|  | *Pomatorhinus erythrocnemis* | Spot-breated Scimitar Babbler | 4 | 1 | 0 | 1 |
|  | *Pomatorhinus ruficollis* | Rufous-necked Scimitar Babbler | 25 | 3（0.17±0.06） | 2（0.13±0.13） | 4 |
|  | *Napothera epilepidota* | Lesser Wren Babbler | 1 | 0 | 0 | 0 |
|  | *Stachyris ruficeps* | Rufous-capped Babbler | 10 | 3（0.30±0.10） | 2（0.20±0.20） | 3 |
|  | *Stachyris nigriceps* | Black-headed Babbler | 10 | 6（0.60±0.20） | 4（0.40±0.20） | 5 |
|  | *Stachyris striolata* | Spot-necked Babbler | 1 | 1 | 0 | 1 |
|  | *Macronous gularis* | Striped Tit Babbler | 4 | 1 | 2 | 1 |
|  | *Leiothrix argentauris* | Silver-eared Mesia | 6 | 0 | 3 | 2 |
|  | *Leiothrix lutea* | Red-billed Leiothrix | 30 | 2（0.08±0.06） | 2（0.03±0.03） | 4 |
|  | *Pteruthius xanthochlorus* | Green Shrike Babbler | 1 | 0 | 0 | 0 |
|  | *Pteruthius melanotis* | Black-eared Shrike Babbler | 1 | 0 | 1 | 0 |
|  | *Actinodura egertoni* | Rusty-fronted Barwing | 2 | 0 | 1 | 1 |
|  | *Minla cyanouroptera* | Blue-winged Siva | 11 | 0（0.00） | 6（0.25±0.25） | 3 |
|  | *Minla strigula* | Bar-throated Minla | 4 | 0 | 0 | 0 |
|  | *Minla ignotincta* | Fire-tailed Minla | 5 | 0 | 1 | 1 |
|  | *Alcippe castaneceps* | Chestnut-headed Fulvetta | 2 | 1 | 0 | 1 |
|  | *Alcippe dubia* | Rusty-capped Fulvetta | 4 | 0 | 0 | 0 |
|  | *Alcippe brunnea* | Gould's Fulvetta | 18 | 0（0.00） | 0（0.00） | 0 |
|  | *Alcippe poioicephala* | Brown-cheeked Fulvetta | 5 | 0 | 4 | 3 |
|  | *Alcippe morrisonia* | Grey-cheeked Fulvetta | 125 | 4（0.05±0.03） | 33（0.25±0.16） | 11 |
|  | *Alcippe nipalensis* | Nepal Fulvetta | 9 | 0（0.00） | 1（0.13±0.13） | 1 |
|  | *Heterophasia melanoleuca* | Black-headed Sibia | 9 | 2（0.22） | 7 (0.78) | 4 |
|  | *Yuhina castaniceps* | Striated Yuhina | 6 | 0 | 0 | 0 |
|  | *Yuhina flavicollis* | Yellow-napped Yuhina | 11 | 0 (0.00) | 4 (0.38±0.22) | 4 |
|  | *Yuhina gularis* | Stripe-throated Yuhina | 15 | 0 (0.00) | 0 (0.00) | 0 |
|  | *Yuhina nigrimenta* | Black-chinned Yuhina | 4 | 0 | 0 | 0 |
|  | *Erpornis zantholeuca* | White-bellied Yuhina | 6 | 1 | 2 | 2 |
| Panuridae | *Paradoxornis gularis* | Grey-headed Parrotbill | 2 | 1 | 0 | 1 |
|  | *Paradoxornis webbianus* | Vinous-throated Parrotbill | 1 | 0 | 0 | 0 |
|  | *Suthora poliotis* | Grey-breasted Parrotbill | 5 | 0 | 1 | 0 |
|  | *Paradoxornis verreauxi* | Golden Parrotbill | 3 | 0 | 0 | 0 |
| Sylviidae | *Phylloscopus maculipennis* | Ashy-throated Warbler | 4 | 0 | 0 | 0 |
|  | *Phylloscopus proregulus* | Pallas's Leaf Warbler | 1 | 0 | 0 | 0 |
|  | *Phylloscopus borealis* | Arctic Warbler | 1 | 0 | 0 | 0 |
|  | *Phylloscopus reguloides* | Blyth's Leaf Warbler | 6 | 1 | 2 | 1 |
|  | *Seicercus tephrocephalus* | Grey-crowned Warbler | 1 | 0 | 0 | 0 |
|  | *Seicercus valentini* | Bianchi’s Warbler | 5 | 0 | 0 | 0 |
|  | *Seicercus affinis* | White-spectacled Warbler | 2 | 0 | 0 | 0 |
|  | *Seicercus poliogenys* | Grey-cheeked Warbler | 1 | 0 | 0 | 0 |
|  | *Abroscopus superciliaris* | Yellow-bellied Warbler | 1 | 0 | 1 | 1 |
|  | *Abroscopus schisticeps* | Black-faced Warbler | 1 | 0 | 0 | 0 |
| Zosteropidae | *Zosterops japonicus* | Japanese White-eye | 2 | 0 | 0 | 0 |
| Paridae | *Parus monticolus* | Green-backed Tit | 7 | 2 (0.29±0.04) | 0 (0.00) | 1 |
|  | *Parus spilonotus* | Yellow-cheeked Tit | 13 | 0 (0.00) | 3 (0.15±0.15) | 1 |
|  | *Sylviparus modestus* | Yellow-browed Tit | 1 | 0 | 0 | 0 |
| Sittidae | *Sitta nagaensis* | Chestnut-vented Nuthatch | 3 | 0 | 0 | 0 |
| Dicaeidae | *Dicaeum ignipectus* | Fire-breasted Flowerpecker | 1 | 0 | 0 | 0 |
| Nectariniidae | *Hypogramma hypogrammicum* | Purple-naped Sunbird | 1 | 0 | 1 | 1 |
|  | *Aethopyga gouldiae* | Gould’s Sunbird | 4 | 0 | 0 | 0 |
|  | *Aethopyga saturata* | Black-throated Sunbird | 3 | 0 | 0 | 0 |
|  | *Arachnothera longirostra* | Little Spiderhunter | 8 | 0 (0.00) | 4 (0.50) | 2 |
|  | *Arachnothera magna* | Streaked Spiderhunter | 3 | 1 | 0 | 0 |
| Fringillidae | *Pyrrhula nipalensis* | Brown Bullfinch | 2 | 0 | 0 | 0 |
| 26 | 124 | 124 | 728 | 57 | 160 | 79 |

* The prevalence (P %) is given in percentages in cases where seven or more individuals were examined.
