# Supplementary material for: Correction: Prevalence Patterns of Avian Plasmodium and Haemoproteus Parasites and the Influence of Host Relative Abundance in Southern China
Source: PLoS One. 2014 Sep 3;9(9):e107826. doi: 10.1371/journal.pone.0107826 (PMC4153723; doi:10.1371/journal.pone.0107826)
Supplement: Table S2 — Summary of lineage parasite information, including Genbank accession numbers. (DOC) [file pone.0107826.s002.doc]

**Table S2.** Summary of lineage parasite information, including Genbank accession numbers.

| **Lineage** | **Parasite genus** | **Host Species** | | **Host Family** | **sites** | **Genbank** |
| --- | --- | --- | --- | --- | --- | --- |
|  |  | **Scientific name** | **Common name** |  |  |  |
| ABSUP01 | *Haemoproteus* | *Abroscopus superciliaris* | Yellow-bellied Warbler | Sylviidae | XHK | KJ145121 |
| ACEGE01 | *Haemoproteus* | *Actinodura egertoni* | Rusty-fronted Barwing | Timaliidae | PS | KJ145102 |
| ALARV04 | *Plasmodium* | *Rhipidura hypoxantha* | Yellow-bellied Fantail | Rhipiduridae | JZS | KJ145063/ DQ659569 |
|  | *Plasmodium* | *Niltava sundara* (2) | Rufous-bellied Niltava | Muscicapidae | DWS、JZS |  |
|  | *Plasmodium* | *Phylloscopus reguloides* | Blyth's Leaf Warbler | Sylviidae | JZS |  |
| ALBRE01 | *Haemoproteus* | *Alophoixus pallidus* (5) | White-throated Bulbul | Pycnonotidae | XHK、HML | KJ145079/ AF465571 |
|  | *Haemoproteus* | *Hypogramma hypogrammicum* | Purple-naped Sunbird | Nectariniidae | XHK |  |
|  | *Haemoproteus* | *Stachyris nigriceps* | Black-headed Babbler | Timaliidae | XHK |  |
|  | *Haemoproteus* | *Terpsiphone paradisi* | Asian Paradise Flycatcher | Monarchidae | XHK |  |
| ALMOR01 | *Plasmodium* | *Heterophasia melanoleuca* | Black-headed Sibia | Timaliidae | JZS | KJ145048/ EF380141 |
| ALMOR03 | *Plasmodium* | *Alcippe morrisonia* | Grey-cheeked Fulvetta | Timaliidae | NL | KJ145071 |
| ALMOR04 | *Plasmodium* | *Stachyris ruficeps*（2） | Rufous-capped Babbler | Timaliidae | BDGS | KJ145070 |
|  | *Plasmodium* | *Alcippe morrisonia* | Grey-cheeked Fulvetta | Timaliidae | BDGS |  |
| ALMOR05 | *Haemoproteus* | *Stachyris ruficeps* | Rufous-capped Babbler | Timaliidae | PS | KJ145088 |
|  | *Haemoproteus* | *Alcippe morrisonia* (3) | Grey-cheeked Fulvetta | Timaliidae | PS |  |
| ALMOR06 | *Haemoproteus* | *Cinclidium leucurum* (2) | White-tailed Blue Robin | Turdidae | XHK、PS | KJ145089 |
|  | *Haemoproteus* | *Arachnothera longirostra* | Little Spiderhunter | Nectariniidae | XHK |  |
|  | *Haemoproteus* | *Pellorneum albiventre* | White-bellied Jungle Babbler | Timaliidae | XHK |  |
|  | *Haemoproteus* | *Alcippe poioicephala* (2) | Brown-cheeked Fulvetta | Timaliidae | XHK |  |
|  | *Haemoproteus* | *Alcippe morrisonia* | Grey-cheeked Fulvetta | Timaliidae | XHK、PS |  |
|  | *Haemoproteus* | *Ficedula monileger* | White-gorgetted Flycatcher | Muscicapidae | XHK |  |
| ALMOR07 | *Haemoproteus* | *Alcippe morrisonia* | Grey-cheeked Fulvetta | Timaliidae | PS | KJ145114 |
| ALMOR08 | *Haemoproteus* | *Alcippe morrisonia* (2) | Grey-cheeked Fulvetta | Timaliidae | XHK、PS | KJ145103 |
| ALMOR09 | *Haemoproteus* | *Hypsipetes mcclellandii* | Green-winged Bulbul | Pycnonotidae | PS | KJ145120 |
|  | *Haemoproteus* | *Alcippe morrisonia* | Grey-cheeked Fulvetta | Timaliidae | PS |  |
|  | *Haemoproteus* | *Parus spilonotus* | Yellow-cheeked Tit | Paridae | PS |  |
|  | *Haemoproteus* | *Rhipidura albicollis* | White-throated Fantail | Rhipiduridae | PS |  |
| ALMOR10 | *Haemoproteus* | *Alcippe morrisonia* | Grey-cheeked Fulvetta | Timaliidae | PS | KJ145124 |
|  | *Haemoproteus* | *Yuhina flavicollis* | Yellow-napped Yuhina | Timaliidae | PS |  |
|  | *Haemoproteus* | *Minla cyanouroptera* | Blue-winged Siva | Timaliidae | PS |  |
| ALNIP01 | *Haemoproteus* | *Alcippe nipalensis* | Nepal Fulvetta | Timaliidae | PS | KJ145117 |
| ALPAL01 | *Haemoproteus* | *Alophoixus pallidus* | White-throated Bulbul | Pycnonotidae | HML | KJ145072 |
| ALPAL02 | *Haemoproteus* | *Alophoixus pallidus* | White-throated Bulbul | Pycnonotidae | XHK | KJ145084 |
| ALPOI01 | *Haemoproteus* | *Alcippe morrisonia* | Grey-cheeked Fulvetta | Timaliidae | XHK | KJ145086 |
|  | *Haemoproteus* | *Pellorneum albiventre* | White-bellied Jungle Babbler | Timaliidae | XHK |  |
|  | *Haemoproteus* | *Alcippe poioicephala* | Brown-cheeked Fulvetta | Timaliidae | XHK |  |
| ALPOI02 | *Haemoproteus* | *Alcippe poioicephala* | Brown-cheeked Fulvetta | Timaliidae | XHK | KJ145106 |
| ARLON01 | *Haemoproteus* | *Arachnothera longirostra* | Little Spiderhunter | Nectariniidae | XHK | KJ145115 |
| CILEU01 | *Haemoproteus* | *Cinclidium leucurum* | White-tailed Blue Robin | Turdidae | NL | KJ145075 |
| CILEU02 | *Haemoproteus* | *Cinclidium leucurum* | White-tailed Blue Robin | Turdidae | PS | KJ145123 |
| COPMAL01 | *Haemoproteus* | *Copsychus malabaricus* | White-rumped Shama | Turdidae | HML | KJ145083/ AF465587 |
| COPMAL02 | *Haemoproteus* | *Copsychus malabaricus* | White-rumped Shama | Turdidae | HML | KJ145093 |
| CYBAN01 | *Haemoproteus* | *Cyornis banyumas* | Hill Blue Flycatcher | Muscicapidae | XHK | KJ145099 |
| CYHAI01 | *Plasmodium* | *Hypothymis azurea* | Black-naped Monarch | Monarchidae | XHK | KJ145056 |
|  | *Plasmodium* | *Cyornis hainanus* | Hainan Blue Flycatcher | Muscicapidae | XHK |  |
| DEFOR01 | *Haemoproteus* | *Dendrocitta formosae* | Gray Treepie | Corvidae | HML | KJ145082 |
| DELURB5 | *Plasmodium* | *Heterophasia melanoleuca* | Black-headed Sibia | Timaliidae | JZS | KJ145049/ EU154347 |
|  | *Plasmodium* | *Parus monticolus* | Green-backed Tit | Paridae | JZS |  |
|  | *Plasmodium* | *Ficedula hyperythra* | Snowy-browed Flycatcher | Muscicapidae | JZS |  |
| ERZAN01 | *Plasmodium* | *Erpornis zantholeuca* | White-bellied Yuhina | Timaliidae | XHK | KJ145059 |
| ERZAN02 | *Haemoproteus* | *Erpornis zantholeuca* | White-bellied Yuhina | Timaliidae | XHK | KJ145105 |
| FIMON01 | *Haemoproteus* | *Ficedula monileger* | White-gorgetted Flycatcher | Muscicapidae | XHK | KJ145109 |
| FIPAR02 | *Haemoproteus* | *Tarsiger cyanurus* | Red-flanked Bush Robin | Turdidae | NL | KJ145077/ EF380197 |
| FIWES01 | *Haemoproteus* | *Ficedula westermanni* | Little Pied Flycatcher | Muscicapidae | JZS | KJ145112 |
| FIZAN01 | *Haemoproteus* | *Ficedula zanthopygia* | Yellow-rumped Flycatcher | Muscicapidae | HML | KJ145100 |
| GLBRO01 | *Haemoproteus* | *Glaucidium brodiei* | Collared Pygmy Owl | Strigidae | NL | KJ145074 |
| GLBRO02 | *Haemoproteus* | *Glaucidium brodiei* | Collared Pygmy Owl | Strigidae | HML | KJ145080 |
|  | *Haemoproteus* | *Copsychus malabaricus* | White-rumped Shama | Turdidae | HML |  |
|  | *Haemoproteus* | *Cyornis hainanus* | Hainan Blue Flycatcher | Muscicapidae | HML |  |
| GRW06 | *Plasmodium* | *Stachyris nigriceps* | Black-headed Babbler | Timaliidae | PS | KJ145065/ DQ368381 |
|  | *Plasmodium* | *Stachyris ruficeps* | Rufous-capped Babbler | Timaliidae | PS |  |
|  | *Plasmodium* | *Alcippe morrisonia* | Grey-cheeked Fulvetta | Timaliidae | NL |  |
| HECAS01 | *Haemoproteus* | *Hemixos castanonotus* | Chestnut Bulbul | Pycnonotidae | NL | KJ145078 |
| HEMEL01 | *Haemoproteus* | *Heterophasia melanoleuca* (6) | Black-headed Sibia | Timaliidae | JZS | KJ145087 |
| HEMEL02 | *Haemoproteus* | *Heterophasia melanoleuca* | Black-headed Sibia | Timaliidae | JZS | KJ145095 |
| HYAZU01 | *Plasmodium* | *Hypothymis azurea* | Black-naped Monarch | Monarchidae | XHK | KJ145061 |
| HYPHI27 | *Haemoproteus* | *Hypsipetes leucocephalus* | Black Bulbul | Pycnonotidae | JZS | KJ145097/ JN975307 |
| HYPRO01 | *Haemoproteus* | *Iole propinqua* | Grey-eyed Bulbul | Pycnonotidae | XHK | KJ145125 |
|  | *Haemoproteus* | *Pellorneum albiventre* | White-bellied Jungle Babbler | Timaliidae | XHK |  |
| LEARG01 | *Haemoproteus* | *Leiothrix argentauris* (2) | Silver-eared Mesia | Timaliidae | PS | KJ145094 |
| LEARG02 | *Haemoproteus* | *Leiothrix argentauris* | Silver-eared Mesia | Timaliidae | PS | KJ145096 |
| LELUT01 | *Haemoproteus* | *Leiothrix lutea* | Red-billed Leiothrix | Timaliidae | NL | KJ145090 |
| LELUT02 | *Haemoproteus* | *Leiothrix lutea* | Red-billed Leiothrix | Timaliidae | NL | KJ145101 |
| MEVIR01 | *Haemoproteus* | *Megalaima virens* | Great Barbet | Capitonidae | BDGS | KJ145073 |
| MICYA01 | *Haemoproteus* | *Minla cyanouroptera* | Blue-winged Siva | Timaliidae | PS | KJ145091 |
| MICYA02 | *Haemoproteus* | *Minla cyanouroptera* | Blue-winged Siva | Timaliidae | PS | KJ145119 |
| MIIGO01 | *Haemoproteus* | *Minla ignotincta* | Fire-tailed Minla | Timaliidae | DWS | KJ145118 |
| MONTRI01 | *Plasmodium* | *Hypothymis azurea* | Black-naped Monarch | Monarchidae | XHK | KJ145054/ AY714199 |
| MUFER01 | *Haemoproteus* | *Muscicapa ferruginea* | Ferruginous Flycatcher | Muscicapidae | JZS | KJ145085 |
| MYCAE01 | *Plasmodium* | *Myophonus caeruleus* | Blue Whistling Thrush | Turdidae | NL | KJ145064 |
| NIGRA01 | *Haemoproteus* | *Niltava grandis* (2) | Large Niltava | Muscicapidae | PS | KJ145104 |
| NILSUN01 | *Plasmodium* | *Niltava sundara* | Rufous-bellied Niltava | Muscicapidae | JZS | KJ145068 |
| NILSUN02 | *Haemoproteus* | *Niltava sundara* (2) | Rufous-bellied Niltava | Muscicapidae | JZS | KJ145111 |
| NILTAV01 | *Haemoproteus* | *Enicurus leschenaulti* | White-crowned Forktail | Turdidae | JZS | KJ145108/ AY714199 |
|  | *Haemoproteus* | *Alcippe morrisonia* (3) | Grey-cheeked Fulvetta | Timaliidae | PS |  |
|  | *Haemoproteus* | *Niltava davidi* (2) | Fujian Niltava | Muscicapidae | PS |  |
|  | *Haemoproteus* | *Niltava sundara* (6) | Rufous-bellied Niltava | Muscicapidae | JZS |  |
| ORW1 | *Plasmodium* | *Pycnonotus sinensis* | Chinese Bulbul | Pycnonotidae | HML | KJ145050/ AF254963 |
|  | *Plasmodium* | *Otus bakkamoena* | Collared Scops Owl | Strigidae | HML |  |
|  | *Plasmodium* | *Zoothera citrine* (2) | Orange-headed Ground Thrush | Turdidae | XHK、HML |  |
| PAGUL01 | *Plasmodium* | *Leiothrix lutea* | Red-billed Leiothrix | Timaliidae | NL | KJ145047 |
|  | *Plasmodium* | *Paradoxornis gularis* | Grey-headed Parrotbill | Panuridae | BDGS |  |
| PIPHA01 | *Haemoproteus* | *Pitta phayrei* | Eared Pitta | Pittidae | XHK | KJ145081 |
|  | *Haemoproteus* | *Niltava macgregoriae* | Small Niltava | Muscicapidae | PS |  |
| POERY01 | *Plasmodium* | *Pomatorhinus erythrocnemis* | Spot-breated Scimitar Babbler | Sylviidae | BDGS | KJ145069 |
| POHYP01 | *Plasmodium* | *Pomatorhinus hypoleucos* | Large Scimitar Babbler | Timaliidae | HML | KJ145053 |
| POMFER01 | *Plasmodium* | *Hypsipetes mcclellandii* | Green-winged Bulbul | Pycnonotidae | PS | KJ145051/ DQ659585 |
|  | *Plasmodium* | *Hypothymis azurea* | Black-naped Monarch | Monarchidae | XHK |  |
|  | *Plasmodium* | *Ficedula monileger* | White-gorgetted Flycatcher | Muscicapidae | XHK |  |
|  | *Plasmodium* | *Cyornis banyumas* | Hill Blue Flycatcher | Muscicapidae | XHK |  |
| PORUF01 | *Plasmodium* | *Pomatorhinus ruficollis* | Rufous-necked Scimitar Babbler | Timaliidae | PS | KJ145060 |
| PORUF02 | *Plasmodium* | *Pomatorhinus ruficollis* | Rufous-necked Scimitar Babbler | Timaliidae | NL | KJ145058 |
| PORUF03 | *Plasmodium* | *Leiothrix lutea* | Red-billed Leiothrix | Timaliidae | BDGS | KJ145067 |
|  | *Plasmodium* | *Macronous gularis* | Striped Tit Babbler | Timaliidae | XHK |  |
|  | *Plasmodium* | *Pomatorhinus ruficollis* | Rufous-necked Scimitar Babbler | Timaliidae | JZS |  |
| PORUF04 | *Haemoproteus* | *Pomatorhinus ruficollis* | Rufous-necked Scimitar Babbler | Timaliidae | PS | KJ145110 |
| PYCGOI01 | *Haemoproteus* | *Pycnonotus sinensis* (2) | Chinese Bulbul | Pycnonotidae | HML | KJ145092/ JX418173 |
| PYFLA01 | *Plasmodium* | *Pycnonotus flavescens* | Flavescent Green Bulbul | Pycnonotidae | PS | KJ145055 |
| STANIG01 | *Plasmodium* | *Stachyris nigriceps* (3) | Black-headed Babbler | Timaliidae | PS | KJ145062/ EF380160 |
| STANIG02 | *Plasmodium* | *Pellorneum albiventre* | White-bellied Jungle Babbler | Timaliidae | XHK | KJ145057 |
|  | *Plasmodium* | *Stachyris nigriceps* (2) | Black-headed Babbler | Timaliidae | XHK |  |
|  | *Plasmodium* | *Alcippe castaneceps* | Chestnut-headed Fulvetta | Timaliidae | PS |  |
| STANIG03 | *Haemoproteus* | *Stachyris nigriceps* | Black-headed Babbler | Timaliidae | XHK | KJ145122 |
|  | *Haemoproteus* | *Chalcophaps indica* | Emerald Dove | Columbidae | XHK |  |
| STSTR01 | *Plasmodium* | *Stachyris striolata* | Spot-necked Babbler | Timaliidae | XHK | KJ145066 |
| TUBOU01 | *Plasmodium* | *Turdus boulboul* | Grey-winged Blackbird | Turdidae | JZS | KJ145052 |
| TYTAL1 | *Haemoproteus* | *Otus bakkamoena*（2） | Collared Scops Owl | Strigidae | HML | KJ145076/ JN863575 |
| YUFLA01 | *Haemoproteus* | *Yuhina flavicollis* | Yellow-napped Yuhina | Timaliidae | DWS | KJ145098 |
| YUFLA02 | *Haemoproteus* | *Yuhina flavicollis* | Yellow-napped Yuhina | Timaliidae | DWS | KJ145107 |
| YUFLA03 | *Haemoproteus* | *Yuhina flavicollis* | Yellow-napped Yuhina | Timaliidae | DWS | KJ145113 |
| ZOOLUN01 | *Haemoproteus* | *Niltava grandis* | Large Niltava | Muscicapidae | PS | KJ145116/ AY714150 |
|  | *Haemoproteus* | *Cyornis banyumas* | Hill Blue Flycatcher | Muscicapidae | XHK |  |
| *Plasmodium_gallinaceum* | *Plasmodium* | *?* | ? | ? | ? | AF069612 |

* Previously described lineages found are mark in underline. Number of infected individuals per site is indicated in parenthesis. Survey sites are coded: BDGS, Badagongshan; NL, Nanling; HML, Houmiling; XHK, Xinhuikuan; JZS, Jizushan; DWS, Daweishan; PS, Pingshan.
